# Facilitators and barriers of mHealth interventions during the Covid-19 pandemic: systematic review

**DOI:** 10.1186/s12913-023-10171-w

**Published:** 2023-10-28

**Authors:** Parastoo Amiri, Hamed Nadri, Kambiz Bahaadinbeigy

**Affiliations:** 1 Department of Health Information Technology, School of Allied Medical Sciences, Lorestan University of Medical Sciences, Khorramabad, Iran; 2 Department of Health Information Technology, , School of Allied Medical Sciences, Urmia University of Medical Sciences, Urmia, Iran; 3 Medical Informatics Research Center, Institute of Futures Studies in Health, Kerman University of Medical Sciences, Kerman, Iran

**Keywords:** Mobile health, Covid-19, Barriers, Facilitators, Systematic review

## Abstract

**Background:**

With the spread of Covid-19 disease, health interventions related to the control, prevention, and treatment of this disease and other diseases were given real attention. The purpose of this systematic review is to express facilitators and barriers of using mobile health (mHealth) interventions during the Covid-19 pandemic.

**Methods:**

In this systematic review, original studies were searched using keywords in the electronic database of PubMed until August 2022. The objectives and outcomes of these studies were extracted. Finally, to identify the facilitators and barriers of mHealth interventions, a qualitative content analysis was conducted based on the strengths, weaknesses, opportunities, and threats (SWOT) analysis method with Atlas.ti 8 software. We evaluated the studies using the Mixed Methods Appraisal Tool (MMAT).

**Results:**

In total, 1598 articles were identified and 55 articles were included in this study. Most of the studies used mobile applications to provide and receive health services during the Covid-19 pandemic (96.4%). The purpose of the applications was to help prevention (17), follow-up (15), treatment (12), and diagnosis (8). Using SWOT analysis, 13 facilitators and 18 barriers to patients’ use of mHealth services were identified.

**Conclusion:**

Mobile applications are very flexible technologies that can be customized for each person, patient, and population. During the Covid-19 pandemic, the applications designed due to lack of interaction, lack of time, lack of attention to privacy, and non-academic nature have not met their expectations of them.

**Supplementary Information:**

The online version contains supplementary material available at 10.1186/s12913-023-10171-w.

## Introduction

Covid-19 disease was first reported on December 29, 2019 in Wuhan (China) and officially this global event was named by the World Health Organization (WHO) as the 2019 coronavirus infectious disease (Covid-19) [1, 2]. This disease spread rapidly throughout the world [3]. To control Covid-19 pandemic, WHO called all medical and non-medical groups to help. Controlling this virus required compliance with health and prevention protocols such as social distancing, travel restrictions, quarantine, and isolation of infected people [4]. Reducing the risk of this virus through social distancing with patients was one of the factors that encouraged the Ministry of Health to use technologies such as mobile health (mHealth) [5–9]. According to the WHO definition, mHealth be called “medical and public health practice supported by mobile devices, such as mobile phones, patient monitoring devices, personal digital assistants (PDAs), and other wireless devices” [10]. There are several studies on the use of mHealth interventions to manage diseases such as HIV, type 2 diabetes and etc., which have shown the effectiveness and feasibility of mHealth interventions [11–13]. Then, mHealth can play an effective role in reducing the threat of the spread of the Covid-19 disease by providing affordable and accessible answers to promote public health [14–16].

It is safe to say that the mHealth revolution happened with the outbreak of the Covid-19 pandemic [17] because mHealth led to the improvement of healthcare services [18]. During Covid-19 pandemic, mHealth was more attractive due to the possibility of remote monitoring, screening, triage, diagnosis, and monitoring by governments, health professionals, and healthcare organizations [19–21]. With the increasing benefits offered by mHealth during the pandemic, at the individual level, the technology has grown in popularity for consumers and patients to manage both their exposure risk and the progression of their symptoms [22, 23]. For example, mobile apps can promote the practice of self-care and enable individual responsibility for disease prevention and health maintenance [22, 24]. mHealth can be used to track any pandemic, as vaccine reminders, to inform people about their health and self-monitoring [25, 26].

During the outbreak of the Covid-19 disease, the mHealth applications (mHealth-app) to improve the health of patients have been shown in several studies [27–30]. A study in China used a mHealth-app to monitor infected people [31]. The system included reports, diagnostic tests, medical records, and social media. In another study, researchers used mHealth-app to effectively reduce the transmission of the Covid-19 disease among people [29]. Also, some mHealth-app that use Bluetooth and Global Positioning System (GPS) features can be useful in Covid-19 contact tracing [32]. A mHealth-app helped improve the blood sugar control of type 2 diabetes patients during the Covid-19 pandemic so that patients can manage their diabetes [2]. Another mHealth-app evaluated safe messages, checking their content and performance in order to encourage AIDS patients to participate in their own care during the spread of the Covid-19 virus [33]. Many studies have shown that governments that have developed mHealth-app in low-income areas, they have indirectly promoted equity in access to healthcare by fixing the existing gaps in limited resources, such as internet access [34, 35].

While the acceptance of these technologies is always increasing [36] but their application faces certain challenges and obstacles [37, 38]. For example, the pattern of using mHealth-app is different among different age groups [39]. In addition, socio-economic factors can be considered among other obstacles in the adoption of mHealth-app [40]. For this reason, mHealth-app adoption may be challenging in developing countries [41]. By examining 339 mHealth-apps in Indonesia, Sujarwoto et al. [42]. showed that the lack of data security and data privacy protection, integration, and infrastructure were the main challenges of Covid-19 apps. Based on our studies, the available review articles on mHealth-app in the context of Covid-19 focus on specific topics such as cancer and aging [43, 44]. Also, based on our knowledge, no study was found that generally examines the facilitators and challenges of implementing mHealth-app interventions for all patients during the Covid-19 pandemic. Also, the implementation of mHealth interventions has been less analyzed to date. The complexity of its implementation hindered the adaptability of the mHealth interventions. Therefore, the purpose of this study is to identify facilitators and barriers of implementing mHealth interventions for patients during the outbreak of the Covid-19 pandemic.

## Methods

The present systematic review is reported based on Preferred Reporting Items for Systematic reviews and Meta-Analyses (PRISMA) [45]. The PubMed database was searched for relevant articles published in English up to August 2022. For this purpose, we used a combination of the keywords of Covid-19 disease and mHealth in the title and abstract (Table 1).

**Table 1:** Groups of keywords used in the search strategy

Group 1	“COVID-19” OR “COVID19” OR “Coronavirus” OR “SARS-CoV-2”
Group 2	“Mobile health” OR “mHealth” OR “Smart-phone” OR “Mobile phone” OR “Mobile applications” OR “Mobile apps”

*MeSH terms are in bold

### Study eligibility criteria

The inclusion and exclusion criteria were then applied to the included articles.

### Inclusion criteria

Articles with the following attributes were included in the review:

1. Articles were written in English.
2. Investigating facilitators and barriers of mHealth interventions.

### Exclusion criteria

The following types of articles were excluded:

1. Did not specifically focus on mHealth interventions.
2. Articles that do not address barriers or facilitators of mHealth interventions.
3. Non-mHealth interventions that do not have a mobile base (telemedicine, other types of eHealth and use of other telecommunication technologies, such computers, internet or e-mail).
4. Review articles, letters to the editor, conference abstracts, and author comments were excluded from the study.

### Quality assessment of study methodology

We utilized the Mixed Methods Appraisal Tool (MMAT) to evaluate the methodological quality of the studies included in our review [46]. The MMAT is specifically created to allow researchers to assess the quality of quantitative, qualitative, and mixed-methods research concurrently, enabling the comparison of scores across various study designs. The assessment of study quality is determined by dividing the number of criteria met by the total relevant criteria within each domain. The application of MMAT consistently yielded strong inter-class correlation, with values ranging from 0.84 to 0.94 [46]. Two independent reviewers (PA and HN) conducted separate assessments and computed scores for each study. Any disagreements between the reviewers were resolved through discussion, followed by a reevaluation of the studies.

### Review process

**Fig. 1:**
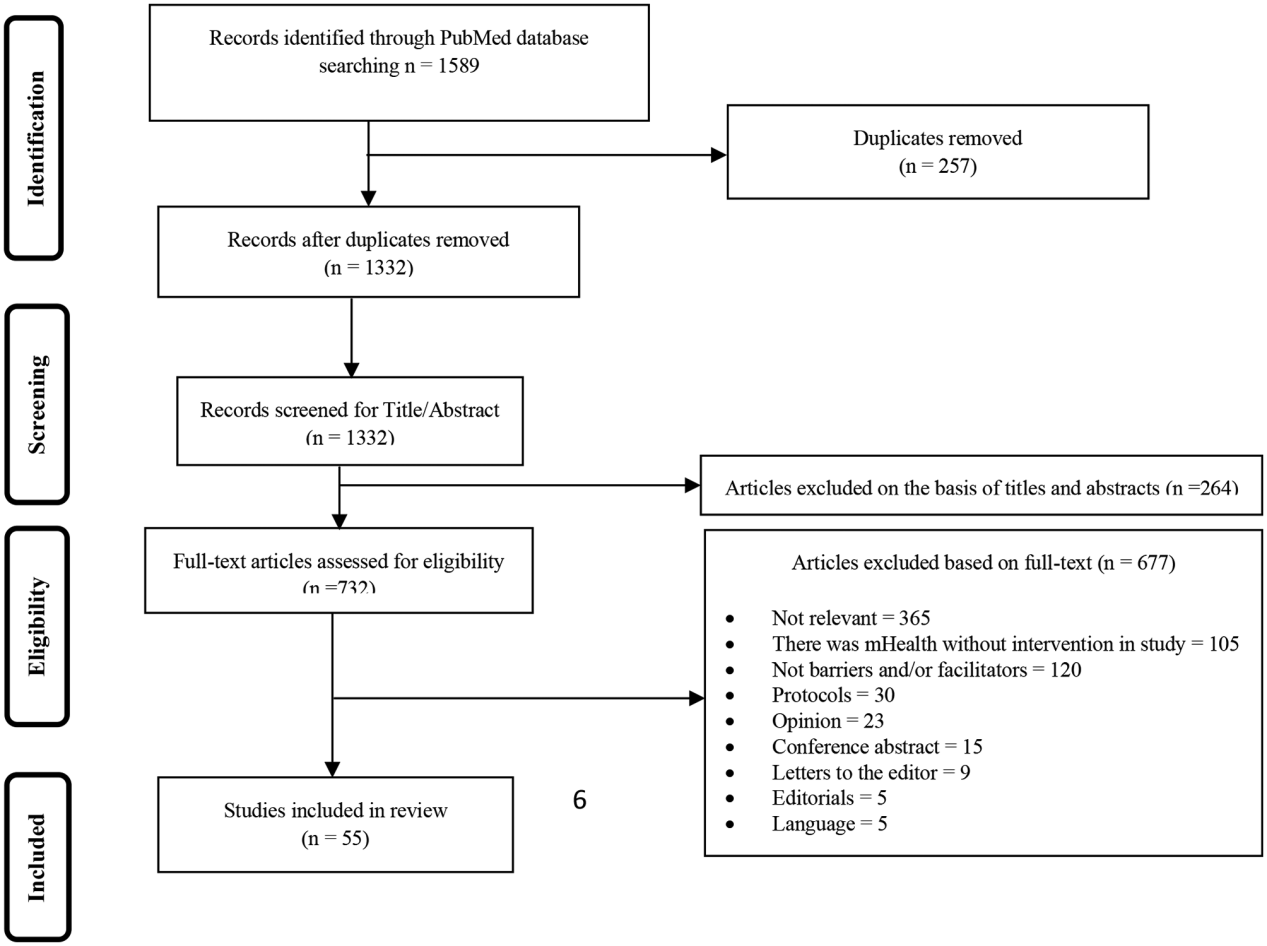
Flow diagram of study selection

Figure 1 shows the flow diagram of the review process. Three authors independently screened the titles and abstracts of the articles to find other relevant studies based on the inclusion and exclusion criteria. Relevant articles were selected for a full-text review, and disagreements were resolved by consensus. A data list of the selected full-text articles was generated in the Excel Spreadsheet software version 2018.

### Data extraction and analysis

**Fig. 2:**
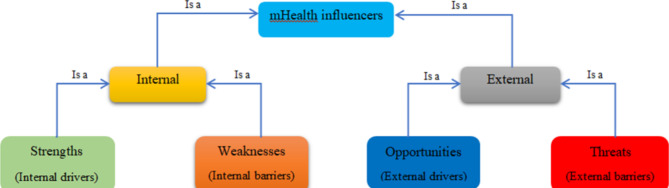
The influence of facilitators and barriers to the use of mobile health interventions during the Covid-19 pandemic

In the present study, the facilitators and barriers in mHealth interventions were identified by qualitative content analysis in Atlas.ti software version 8.4.24 [47]. Then they were collected in four main groups including Strengths, Weaknesses, Opportunities, and Threats (SWOT) in Fig. 2. SWOT analysis enables the identification of internal and external factors affecting the performance of a technology. Also, this analysis is one of the main tools used to inform decision-makers about the effectiveness of technology [48]. For this reason, we considered it a suitable tool for the strategic evaluation of mHealth interventions:

Strengths: Internal factors related to incentives and facilitators of mHealth interventions, such as the positive feedback, comments, factors, and indicators that affect the behavior and motivation of the individual while using the system.

Weaknesses: Internal factors related to the limitations and challenges of mHealth interventions, such as the negative feedback, opinions, factors, and indicators regarding the surrounding environment of applying the system and the system itself.

Opportunities: External factors related to incentives and facilitators of mHealth interventions, such as the positive feedback, opinions, factors, and indicators that affect the behavior and motivation of the individual while using the system.

Threats: External factors related to the limitations and challenges of mHealth interventions, such as negative feedback, opinions, factors, and indicators regarding the surrounding environment of applying the system and the system itself.

Two authors (PA and HN) analyzed all the articles and by reading the full text of the article, they extracted the facilitators and barriers in mHealth interventions. After extracting facilitators and barriers (codes), they were grouped into four subthemes of strengths, weaknesses, opportunities, and threats. Then the subthemes were placed in themes (internal and external). Four group meetings were held to discuss the themes and data discovered until reaching an agreement among all the authors. Another author (KB) verified the research results. The authors also extracted basic characteristics from each study, summarized their objectives, and described the mHealth interventions used in each study. In addition, they summarized the effectiveness of the mHealth interventions used in each article.

## Results

In general, 1589 articles were extracted. After removing duplicate articles, the title and abstract of 1332 articles were reviewed. Of these, 732 articles were reviewed for further review based on their entire text which finally included 55 articles (Fig. 1). Details of the articles are provided in supplementary file 1. 46 of 55 studies met all MMAT criteria (supplementary file 2).

### Details of included studies

In supplementary file 1, the articles are listed in order of publication year from 2022 to 2020. Most of the articles (24 articles (43.6%)) were related to 2021. The majority of studies were related to the United States. Of all the articles, only 15 articles (27.3%) were related to Asia (China, Taiwan, South Korea, Thailand, Iran, Turkey, Saudi Arabia, Indonesia, Singapore, Afghanistan, and Jordan).

Of the 55 related articles found, 41 were quantitative, nine were qualitative, and five were mixed (supplementary file 1). Of all quantitative articles, 12 articles (21.82%) were RCT type. Most of the studies (34 articles (61.82%)) were related to academic centers. In 20 articles (36.4%), patients or their caregivers received healthcare services in their home environment. In another 35 articles (63.7%), the place of receiving healthcare services for patients and their caregivers was an equipped room in hospitals, universities, rural clinics, outpatient clinics, or other medical service centers.

### Participants

The participants in the studies found were people with any type of disease who had used mHealth interventions to improve their disease during the Corona crisis (41.82%). In some studies, the participants were healthy people at risk of anxiety or depression due to Covid-19 quarantine (32.73%), healthcare providers (18.2%), and pregnant women and mothers (7.3%).

### Duration of studies

In the studies included in the current study, the duration of the studies varied between days, weeks, months, and years. The minimum duration of the study was reported to be two days and the maximum duration of the study was three years. In five studies, the duration of the studies was not stated.

### Technology intervention

In the studies included in this study, applications and SMS were used to health services. Most of the studies included in this research used mobile applications during the Corona crisis (96.4%). This means that they had installed an application on the mobile phone to provide and receive health services. But in two articles SMS was used to provide and receive health services. Almost half of the applications used in the articles needed the Internet to communicate (36.4%), except for the articles that used SMS.

### Type of intervention technology

In the studies included in this study, most of the applications were researcher-made (81.8%) and some were pre-made (18.1%). In the applications developed by the researcher, 71% were independent and 29% depended on such technologies (electronic health records, hospital information systems, and registries). All researcher-made applications were also independent, except for one application that was dependent on the hospital information system.

### Technology purpose

Of the 55 included studies, mHealth-app was used in most of them (53 studies). In only two studies, one for prevention and the other for follow-up, SMS was used as a technology. In 53 studies with mHealth-app, the application used in 17 studies was to help prevention, 15 studies were related to follow-up, 12 studies were related to treatment, and eight studies were related to disease diagnosis. In only one study was the mHealth-app used for both prevention and follow-up of the disease.

### Objectives of studies

The 55 studies included in this research had various objectives. Among them, eight studies pursued multiple objectives. In most of the studies (18 articles), the effectiveness of implementing mHealth-app to support patients was investigated. 9 studies dealt with how to develop mHealth-app. In addition to the mentioned objectives, in some studies, these applications were mentioned as tracking, support, measurement, prediction, self-care, self-management, and monitoring tools to improve the condition of patients (20 articles). Other studies that used the mHealth-app had objectives such as design, follow-up, evaluation, etc. However, in two studies that used SMS, one aimed to evaluate the barriers to the implementation of mobile health and the other was to evaluate the security of SMS for patients.

### Outcomes

In the included studies, the outcomes were divided into four categories: clinical outcomes, social outcomes, increased quality of life, and satisfaction of patients and health service providers. Out of 55 articles included in this study, 26 studies (47.27%) mentioned the clinical outcomes of using mobile technology. These technologies had led to the improvement of their health by meeting some of the treatment needs of the patients. Also, in 22 studies (40%), the social outcomes of the use of these technologies were mentioned, such as speeding up the control of Covid-19 disease, speeding up the identification of suspected Covid-19 patients, etc. Six studies (10.9%) indicated an increase in the satisfaction of patients and health service providers, and five studies (9.1%) indicated an increase in quality of life. They expressed the increase in the quality of life by providing education and psychological support to people with access to prevention and treatment information.

### Facilitators

In 53 included studies, several facilitators for using mobile technologies were mentioned (Fig. 2; Table 2). The main facilitators in the included studies related to improving clinical and social outcomes, increasing the quality of life, and satisfaction of patients and health service providers were mentioned. But in addition to them, other facilitators were also extracted from these studies. The most common of these facilitators included involving patients and their families in improving their health (16 articles (29.1%)), helping to reduce the mental burden caused by the spread of Covid-19 (11 articles (20%)), and increasing the participation of healthcare providers in improving the health of patients (7 articles (12.7%)).

**Table 2:** SWOT analysis of included studies

**Strengths**
Ease of use: A general concept that refers to the simplicity such as tracking physical activity and medication reminders
Collaboration: A spectrum of interactions to enhance healthcare services and improve patient outcomes
User-friendliness: A general concept that refers to clear, simple and quick access to information without unnecessary steps or confusion
Improve delivery of services: Offer easy appointment scheduling and reminders through mHealth intervention
Access to medical/health information at the point-of-care: To access up-to-date and comprehensive information to support clinical decision-making, diagnosis, and treatment
Familiarizing users with mobile phone services: To empower users with the knowledge and skills to effectively use their mobile phones
Real-time supervision and monitoring: The continuous, immediate, and remote tracking of patient data
**Weaknesses**
Local language: Limited to a specific language
Security concerns: Such as threats to sensitive healthcare information
Privacy concerns: Such as cancellation of authentication processes
Confidentiality concerns: Requiring strong user authentication methods, such as PINs, passwords, biometrics, or two-factor authentication
Increasing phone maintenance costs
High upfront set-up costs
Uncertainty on future changes of costs
Software may not be adaptable or flexible
Software may still subject to human error
**Opportunities**
Saving cost: Reducing the financial burden such as use of telemedicine services and remote monitoring
Saving time: Reducing administrative burdens such as appointment scheduling
Open source programs may support implementation of mHealth in low-resource settings
mHealth projects as an innovative method of data collection
Opportunities to be implemented in different national disease control programs
Decreasing communication gap between health workers, managers and patients
**Threats**
Need to mHealth literacy
Need to smartphone
Need to internet
Decreased motivation
The risk of mobile theft and loss
Added workload
Increasing anxiety
Increasing stress
Increasing concern/worry

### Barriers

In the 52 included studies, several barriers to the use of mobile health technologies were mentioned (Fig. 2; Table 2). The need for hardware, software, and the internet was mentioned in most of the studies (38 articles (69.1%)). Software problems such as software errors and technical problems were mentioned in some studies. Also, concerns regarding information security and privacy were mentioned in 8 articles. One of the important barriers to the successful use of mobile health technology is choosing the right language to provide services to the target society (14 articles (25.5%)).

Finally, the review of the articles in this study showed that facilitators and barriers were categorized into two groups, internal and external (Fig. 2).

## Discussion

This study was conducted to evaluate the mhealth-apps used during the Covid-19 era and highlight the weaknesses and strengths of the applications to inform the strengthening and development of this application available in epidemics.

One of the most accessible remote health tools is mobile applications. According to these mobile applications are side tasks of mobile phones, maybe after phone calls, mobile applications are one of the most accessible health tools. These available applications have many features depending on their functionalities. Tracking, prevention, notifications, guides, diagnosis, self-assessment, monitoring, intervention, consultation, and treatment can all be implemented in one application. According to the studies of Mehraeen et al. [48], one of the 20 technology-based approaches that have been identified for providing health care services during the Corona pandemic is mobile applications, which can include many other identified technologies (such as video visits, use of the Internet of Things, etc.). This importance was increasing strongly during the Covid-19 epidemic (especially in the type of tracking and prevention).

In Fig. 2, the barriers and advantages of applications are briefly shown. From the user’s point of view, there are advantages in the application such as ease of use, cooperation, user-friendliness, improvement of service delivery, access to health information at the point of care, familiarization of users with mobile phone services, timely supervision, and monitoring. The internal obstacles include the language limitation of the application, confidentiality, trust, phone maintenance costs, the uncertainty of future costs, and the inflexibility of the software.

The external opportunities of applications include investment, supporting the implementation of mobile health in low-income environments, saving time, the ability to implement in different countries in the prevention of epidemics, and reducing the communication gap between health workers, managers, and patients. External threats include mobile health literacy, the need for smartphones, the need for the Internet, the risk of mobile theft and loss, additional burdens for employees, increased anxiety and stress, and increased worry.

One of the most important barriers of tracking apps is that it does not show the time the infected person stays in one place and even the time of leaving. For this reason, it has caused wrong results during quarantine. In the studies, what is less visible is the decision support tools, which are rarely implemented. Also, another barrier in the studies is that self-evaluation and using the experiences of others in the improvement of symptoms have been less considered.

Our study shows that the largest number of articles related to 2021 and aimed at tracking, follow-up, and prevention were done at the height of the Covid-19 epidemic and nationwide quarantine. Barriers in most of these studies are related to hardware, the internet, and confidentiality and privacy features. White study [49] mentioned that tracking apps play a smaller role than expected. This is because most countries have chosen programmatic configurations that fail to provide a means to quickly notify users of possible infections while avoiding many false positive reports. At the same time, they should require the protection of people’s privacy. Also, most of the articles in this year, unlike in 2022, which are academically and professionally done with the purpose of research, these non-academic studies were conducted for the control of the disease and were carried out by amateurs.

Perhaps one of the most important limitations regarding the use of mobile applications is the need for a device or hardware with the ability to install the application. This limitation exists in our study, especially in developing countries and places where it is not possible to carry a smartphone due to working conditions. Another limitation is the ability to connect to the Internet because most applications need the Internet to access and send information. One of the most important tools for managing patients with the Coronavirus is video communication, and this video communication requires high-speed Internet, while video visits have concerns about the quality of care and the loss of important information due to the lack of a physical examination, according to Darcourt et al. [50]. This important concern requires the Internet of Things, including the use of sensors and tools to obtain vital patient information, especially the amount of oxygen in the body during remote care in corona patients. According to the studies of Shamsabadi et al. [51], one of the most important technologies in the management of chronic diseases during the Corona epidemic is the Internet of Things, which none of the reviewed studies have used this technology. Our study is consistent with studies conducted in different countries [52, 53].

Most of the studies entered and conducted on mobile applications are related to the diagnosis and tracking of the Covid-19 disease, because in the early days of this epidemic, it caused a large-scale quarantine. Several articles are related to other diseases, including the care of pregnant women, people with diabetes, people with tuberculosis, weight loss nutrition, breastfeeding, skin cancer, addiction, AIDS, and physical disabilities. These applications have their limitations, such as the quality of the camera of the mobile device, the speed of the Internet, access to the existing infrastructure related to the medical record, and also the two-way communication with the relevant center for follow-up and consultation. Also, it was not possible to synchronize and integrate with other applications, although they were storing, sending, and aggregating data, a problem that is well shown in Sujarwoto’s study [52]. In most of them, the limitations of authentication and confidentiality have been considered. One of the important advantages of interventions related to disabilities, addiction, and AIDS is involving the user’s family. Family involvement may affect the results of application use [54].

In addition to the physical effects of the Covid-19 disease, there were mental and psychological effects before and after the disease. In this study, there are several articles related to intervention in reducing stress and pressure caused by Covid-19 among treatment workers and people, which only play an advisory role and create guidelines.

An interesting issue in applications that provide people with updated information and guides about Covid-19 is that there is no scoring by users to evaluate and widely use the guide or score, and this is a significant weakness in this type of application. The lack of prescription and drug consumption apps among apps is well felt during the Covid-19 pandemic; while pharmaceutical interventions are one of the most important issues to electronic health interventions [55]. This can be a great help in reducing the spread of epidemics at the international level.

Most of the studies have been conducted on a limited population, and no effort has been made to develop them at the national and international levels. Some applications have been carried out at the level of a small region and even at the level of a university. Another issue with mobile apps is that there are no post-study acceptance evaluations. This can be due to the compulsion to use these applications due to the fear of the Covid-19 epidemic. Tarricone [54] states that durability decreases significantly longitudinally. Therefore, before and during the use of mobile applications that are made due to the spread of Covid-19, the acceptance of the applications should be checked for user acceptance [56].

In most of the reviewed studies, the interoperability between the designed application and infrastructure and electronic records is not considered. According to the authors, it was due to the unexpected spread of Covid-19 and lack of time. Advantages such as the use of FHIR for the ability to interact with the infrastructure of health records and health agencies at the regional and national levels are not clear. Meanwhile, extensive evidence generation is needed to support the integration of mHealth programs in the healthcare field [55, 57].

One of the limitations in our article is that included only one database (PubMed). Of course, our article is not the first study with this limitation. Many studies have also been limited only to searching in PubMed. In tracking studies, one of the limitations is the lack of trust in the results entered into the application by people; because in the case of being sick, there would be restrictions on travel. The results have shown that potential inputs need to be validated by ordinary and related people [55].

## Conclusion

Smartphones are distinctive tools that can be used as very efficient and always up-to-date health devices depending on the capabilities of the built-in applications. This device is very similar to a shell whose content can be changed all the time, although the ability of applications allows for a lot of development. Therefore, with the development of applications in the complex healthcare environment, there are many challenges and advantages that should always be investigated and published. The adaptive nature of applications can be such that it is possible to adapt intervention components for each user and customize them.

### Electronic supplementary material

Below is the link to the electronic supplementary material.

**Supplementary file 1**. Characteristics of included studies in our study

**Supplementary file 2**. Article Quality Assessment based on MMAT

## Data Availability

Our data or material may be available from the first or corresponding author upon reasonable request.
