# Supplementary material for: Facilitators and barriers of mHealth interventions during the Covid-19 pandemic: systematic review
Source: BMC Health Serv Res. 2023 Oct 28;23:1176. doi: 10.1186/s12913-023-10171-w (PMC10613392; doi:10.1186/s12913-023-10171-w)
Supplement: Supplementary file 1 — Characteristics of included studies in our study [file 12913_2023_10171_MOESM1_ESM.docx]

supplementary file 1. Characteristics of included studies in our study

| **Authors**  **(publication year^$^)** | **Country** | **Type of study** | **Healthcare provider setting** | **Patient setting** | **Sample population** | **Study duration** | **Technology intervention** | **App type** | **Technology**  **purpose** | **Study purpose** | **Outcomes** | **drivers** | **Barriers** |
| --- | --- | --- | --- | --- | --- | --- | --- | --- | --- | --- | --- | --- | --- |
| Sun *et al.* (2022) (1) | China | Quantitative/RCT | Academic | University | Individuals (N=114) | 3-month | App | Researcher made/ Standalone | Follow-up | Effectiveness &  Feasibility & Implementation & Acceptability | Cost- effectiveness & Reducing anxiety and  depression among young adults in quarantine & Increasing mindfulness and social support | Reducing the burden of psychological distress | Need to hardware (e.g. smartphone) & Need to software (e.g. Zoom, WeChat) & Need internet |
| Htet *et al.* (2022) (2) | Thailand | Quantitative/Usability study | Academic | University | Patients with tuberculosis (N=453) | 3-month | App | Researcher made/ Standalone | Diagnosis | Usability | Identification of TB patients &  Identification of active TB cases | Involving patient’s family | Need to hardware (e.g. Android phones) & Need internet |
| Yoo *et al.* (2022) (3) | Canada | Quantitative/Retrospective study | Academic | Clinic | Patients with disabilities (N=3124) | 2-year | App | Premade/ Standalone | Prevention | Effectiveness | The expected decrease during the first wave of the Covid-19 pandemic & To help parents, rehabilitation professionals, educators, and communities connect | Involving patients & Involving patient’s family & Free-of-charge & To help them participate in society | Need to hardware (e.g. Android phones) & Need internet |
| Ha *et al.* (2022) (4) | South Korea | Quantitative/RCT | Academic | Hospital | Nurses  (N=60) | 12-week | App | Researcher made/ Standalone | Prevention | Development and effectiveness | Promoting physical activity and sleep quality for nurses | Involving HCWs & Satisfaction | Need to hardware (e.g. smartphone) & Need to software (e.g. Zoom) & Need internet |
| Hodges *et al.* (2022) (5) | USA | Quantitative/Cohort & Pilot study | Academic | Clinic | Addict  (N=25) | 6-month | App | Researcher made/ Standalone | Follow-up | Supporting treatment | Acceptable retention in care & Relapse prevention & Opioid‑associated mortality prevention | Improving self-efficacy and self-control | Need to hardware (e.g. smartphone) & Need internet |
| Mazaheri *et al.* (2022) (6) | Iran | Quantitative/RCT | Academic | Home | Women with premenstrual syndrome  (intervention group= 40, control group = 40) | 8-week | App | Researcher made/ Standalone | Treatment | Effectiveness | Increasing the quality of life & Cost- effectiveness & Reducing the PMS' psychological, physical, and behavioral symptoms | Removing restrictions on in-person treatment during the Covid-19 Pandemic &  Increasing access to treatment | Need to hardware (e.g. smartphone) & Need to software (e.g. WhatsAp) & Need internet & Limitation language |
| Sadural *et al.*(2022) (7) | USA | Qualitative /Pilot study | Academic | Clinic | Mother (N= 13) & Stakeholders (N=7) | 2-week | Message | N/A | Follow-up | Evaluating the barriers to mHealth implementation | Technical issues and the lack of resources for troubleshooting problems & Technical barriers to receiving SMS text messages to understanding SMS text message content & Need to digital health literacy & The lack of vendor support in resolving software bugs | Involving patients & Involving HCWs | Need to hardware (e.g. smartphone) & Need internet & Limitation language & Face-to-face assessment |
| Pulik *et al.* (2022) (8) | Poland | Qualitative /Pilot study | Academic | Hospital | Patients with hip arthroplasty (N>4000) | N/M | App | Researcher made/ Standalone | Treatment | Development | Cost- effectiveness & Patient engagement could reduce surgery-related anxiety & Providing substantial support for patients in conditions of limited information | Involving patients & Increasing patient safety & Free-of-charge & Promoting independence | Need to hardware (e.g. Android phones) & Need internet & Limitation language |
| Quifer-Rada *et al.*(2022) (9) | Spain | Qualitative / Observational & Descriptive & Retrospective study | Non-academic | Home | Individuals  (N= 12,092) | 3-year | App | Researcher made/ Standalone | Follow-up | Evaluating the impact of the COVID-19 pandemic on breastfeeding consultations | Mhealth was a useful tool for breastfeeding support and a powerful tool to identify critical issues of breastfeeding | Involving mothers & Free-of-charge & Monitoring breastfeeding & Tracking functions child growth and child's infant stools | Need to hardware (e.g. smartphone) & Need internet & Limitation language |
| Akin-Sari *et al.*  (2022) (10) | Turkey | Qualitative / RCT | Academic | Home | Individuals  (N= 924) | 2-week | App | Premade/ Standalone | Follow-up | Evaluating the effects of mobile-based cognitive training exercises & Effectiveness | Cost- effectiveness & Decreasing Covid-19 distress, depression, anxiety and stress symptoms | Involving patients & Availability & More accessible | Need to hardware (e.g. smartphone) & Need to software (e.g. Facebook and Twitter) & Need internet & Limitation language |
| Casalino *et al.*  (2022) (11) | Italy | Qualitative /Pilot study | Academic | Home | Patients with COVID-19  (N=N/M) | N/M | App | Researcher made/ Standalone | Prevention | Remote measurement of SpO2 | Avoiding the use of dedicated measuring devices (e.g. saturometers, pulse oximeters) for measuring the Spo_2_ | Involving patients & Increasing user experience & Increasing user trust in technology & Increasing the user's sense of security when using technology | Need to hardware with camera (e.g. , smartphone, tablet, laptop or mobile robots) & Need internet & Limitation language |
| Marco-Ahulló *et al.* (2022) (12) | Spain | Quantitative/Pilot study | Academic | Hospital | Patients with paraplegia (N=14) | 10-month | App | Researcher made/ Standalone | Follow-up | Encouraging people with spinal cord injury to take part in physical activity | Increasing the quality of life & Promoting physical activity levels & Improving the physical and psychological variables of people with spinal cord injury confined to wheelchairs | Increasing physical capacity | Need to hardware (e.g. Android phones and smartwatch) & Full-time use of smartphone & Limitation language |
| Alsaqer *et al.* (2022) (13) | Jordan | Quantitative/RCT & pilot study | Academic | Hospital | 110 older adults with hypertension (intervention group= 40, control group = 80) | 3-month | App | Premade/ Standalone | Prevention | Self-care | Decreasing in systolic blood pressure & improving in self-care maintenance, monitoring, and confidence | Better self-report & Monitoring & Confidence self-care & Improving in the RF, RE, Pain, EF, EW, and SF of QoL | Need to hardware (e.g. Android phones) & Need internet & Limitation language |
| Gonzalez-Ramirez *et al.* (2022) (14) | Spain | Quantitative/RCT & pilot study | Non-academic | Hospital | 104 participants (intervention group= 55, control group = 49) | 4-week | App | Researcher made/  N/M | Prevention | To evaluate the nutrition app to improve dietary profile | To increase carbohydrate intake & Decrease total fat intake &  Support nutrition counseling in primary healthcare | Improving dietary habits | Need to hardware (e.g. smartphone) & Need to software (e.g. nutrition apps) & Limitation language |
| Tseng *et al.* (2022)  (15) | Taiwan | Quantitative/ Development study | Academic | Hospital | Patients in outpatient and emergency department (N=N/M) | 24-month | App | Researcher made/ Integrated | Prevention | Development | To enable patients to access, store, and manage their cross-hospital PHR data | To protect patients’ privacy and data security | Need to hardware (e.g. Android phones) & Need internet & Limitation language |
| Radotra *et al.* (2022)  (16) | England | Qualitative/ Cross-sectional | Non-academic | Home | Plastic surgeons and trainees (N=100) | N/M | App | Researcher made/ Standalone | Treatment | Use of medical photography for peri-operative management of open fractures | Wide spreading utilization of smartphone-based photography to improve patient care at plastic surgery | Improving imaging using a mobile app to help plastic surgeons in the time of Covid-19 | Low image quality compared to professional cameras |
| Alanzi *et al.* (2022) (17) | Saudi Arabia | Quantitative /Cross sectional | Non-academic | Home | Individuals (N=2542) | 8-week | App | Researcher made/ Standalone | Follow-up | To evaluate the mobile app | The application had beneficial effects in reducing the transmission of Covid-19 | Ease of use & Convenience in accessing healthcare services & Access to healthcare information & Good interface & Relevance of tasks and functions on the application | Experienced problems while using the application |
| Gonzalez-Plaza *et al.*(2022) (18) | Spain | Quantitative / RCT | Non-academic | Home | Pregnant women (N=150) | 19-month | App | Researcher made/ Standalone | Treatment | Effectiveness | To increase in physical activity during pregnancy | Safety of the use of a smart band and an app & Support from a midwife during pregnancy & Ease of use of the chat & Utility of pregnancy advice | N/M |
| Jobbágy *et al.* (2022) (19) | Hungary | Quantitative /Retrospective single-center study | Non-academic | Home | Patients with lesions (N=1447) | 4-month | App | Researcher made/ Standalone | Diagnosis | Effectiveness | To provide effective skin cancer care & To reduce of the burden of the health system by minimizing outpatient visits & To decrease the risk of acquiring Covid-19 infection | Fast and accurate triage system effective skin cancer care | No dermoscopy images could be taken during submission of the cases & The quality of the photographs could be highly variable as they were provided by the patients, Important outcomes such as satisfaction and costs were not addressed in this study & Objective inclusion criteria were not determined for the quality of the photographs and it was decided by the dermatologist |
| Gasteiger *et al.* (2022) (20) | New Zealand | Quantitative /prospective cohort | Non-academic | Home | Individuals (N=373) | 2-month | App | Researcher made/ Standalone | Follow-up | Tracking | Contact tracing is a crucial process for controlling and containing infectious diseases | The perceived risk of contracting Covid-19 & Government recommendations and communications & The importance of contact tracing | Technical issues, privacy and security concerns & Forgetfulness & The lack of support from businesses |
| Fukuti *et al.* (2021) (21) | Brazil | Quantitative /Cross- sectional | Non-academic | Clinic | HCWs  (N=913) | 20-week | App | Researcher made/ Standalone | Treatment | Screening | To protect against greater mental stress | Screening, referral, and to offer preventative information (such as videos) & Increasing the motivation of HCWs | N/M |
| Hansen *et al.*(2021) (22) | Denmark | Qualitative/ Cross-sectional | Academic | University | Individuals  (N=23) | 3-week | App | Researcher made/ Standalone | Prevention | Examining user perspectives | Help people to manage and monitor their condition | Involving patients & Increasing motivation & Facilitate social interaction & Sharing of personal health data | Acceptability depends on the adaption of a personalized and user-friendly design & Safety and data security |
| Mbiine *et al.*(2021) (23) | Africa | Quantitative/ Pilot Study | Academic | Hospital | Individuals  (N=20) | One month | App | Researcher made Standalone | Diagnosis | Describing an warning app for screening & Feasibility | User-friendliness of the app among health care workers | Predicting persons likely to have contracted Covid-19 | Need to hardware (e.g. Android phones) & Need internet |
| Golden *et al.*(2021) (24) | USA | Quantitative/ Pilot Study | Academic | Hospital | HCWs  (N=173) | 5-month | App | Researcher made/ Standalone | Prevention | Prediction | Users' need for psychological support | Supporting the mental health of users & Facing fears and active coping & Social support | Data Privacy and security & Need to hardware (e.g. iOS or Android phones) |
| Echeverría *et al.* (2021) (25) | Spain | Quantitative/ Cross-sectional | Non-academic | Clinic | Individuals  (N=76) | 7-month | App | Researcher made/ Standalone | Diagnosis | To minimize the risk of transmission of COVID-19 infection | Assist in early detection and rapid activation of protocols in the workplace | Limiting the risk of spreading the virus & Reducing the economic impact caused by Covid-19 & Help design new strategies for the control Covid-19 | Need to hardware & Privacy |
| Magnani *et al.* (2021) (26) | Pennsylvania | Quantitative/RCT | Academic | Clinic | Patients with atrial fibrillation  (N= 130) | One year | App | Researcher made/ Standalone | Prevention | Effectiveness | Anticoagulation adherence & Patient-centered outcomes & Health care utilization | Eliminate geographic barriers & Potential to enhance digital health access & Advance rural health equity &  To enhance care for geographically remote patients | Need to hardware (e.g. smartphone) |
| Hochstatter *et al.* (2021) (27) | USA | Quantitative/ Pilot Study | Academic | Clinic | People living with HIV and substance use disorder  (N= 64) | One year | App | Researcher made/ Standalone | Prevention | Effectiveness | Increasing the use of illicit substance and contact with other substance-using individuals and reducing their confidence to stay sober and attend recovery meetings | Unfolding drug-related and HIV antiretroviral therapy non-adherence risks such as overdose, unsafe sexual behaviors, and transmission of infectious diseases | Need to hardware (e.g. smartphone) |
| Hanson *et al.* (2021) (28) | UK | Quantitative/Cohort study | Academic | Hospital | Individuals  (N=62) | 9-month | App | Premade/ Integrated | Prevention | Feasibility and effectiveness | Decreasing body weight and improvement in HbA1c | Involving patients & Increasing motivation & Improving self-esteem and self-confidence & Participation in health tracking | Need to hardware (e.g. iOS, Android, web, smartwatch, smart speaker, and virtual reality platforms) & Limitation language |
| Moulaei *et al.* (2021) (29) | Iran | Quantitative/Descriptive study | Academic | Home | Pregnant women  (N=36) | One week | App | Researcher made/ Standalone | Prevention | Development and usability | Reduce maternal anxiety and stress about Covid-19 & Allow quick diagnosis of Covid-19 & Reduce the possibility of infection & Provide rapid access to reliable answers for possible questions & Identify high-risk locations in every city of Iran & Provide pregnant women with instant access to Covid-19 healthcare centers and information about coronavirus & Explain self-care and self-management processes | Involving patients & Monitoring health & Help control and prevent Covid-19 | Need to hardware (e.g. Android phones) & Being literate & Using smartphones daily |
| Anyanwu *et al.*  (2021) (30) | USA | Quantitative/Implementation study | Academic | Hospital | Clinicians  (N= 1104) | 6-month | App | Researcher made/ Standalone | Prevention | Feasibility & Development | Rapid access of physicians to evolving organizational policies and protocols & facilitating remote patient care | Involving HCWs | Need to hardware (e.g. iOS or Android phones) & Need to the hospital intranet & Need to information technology team & Privacy and security |
| Siregar *et al.*(2021) (31) | Indonesia | Mixed-methods/ Development study | Academic | Clinic | Individuals  (N= 442) | 3-month | App | Researcher made/ Standalone | Diagnosis | Screening | Increasing awareness and informative & Early detection of CVD risk & Improving to self-screening tools | Involving patients & Involving HCWs & Awareness of your health status | Need to hardware (e.g. smartphone) & Need to software (e.g. WhatsAp) & Need internet |
| Marshall *et al.*  (2021) (32) | Australia | Quantitative/Pilot study | Academic | University | Individuals  (N= 39) | 6-month | App | Premade/ Standalone | Treatment | Effectiveness | Cost- effectiveness & Increasing the quality of life & Improving in their symptoms of depression, anxiety and distress | Involving patients & Improving the mental health | Need to hardware (e.g. smartphone or tablet device) & Need to software & Need internet & Limitation language |
| Vilendrer *et al.* (2021)  (33) | USA | Mixed-methods/ Implementation study | Academic | Home | Patients with COVID-19 (N=17) | 6-month | App | Researcher made/ Integrated | Prevention | Development | The provision of disease-specific information & Access to testing services | Involving patients & Awareness of your health status | Need to hardware (e.g. Android phones) & Need internet & Limitation language |
| Montanari Vergallo *et al.* (2021)  (34) | Italy | Qualitative/ Cross-sectional | Non-academic | Home | . Patients with COVID-19  (N= N/M) | N/M | App | Researcher made/ Standalone | Follow-up | Tracking | Identify potentially infected individuals before symptoms onset & Piece together the chain of infections in order to break it & Stave off the formation of new infection transmission chains. | Preventing the spread of covid-19 among the community | Concerns due to the potential privacy and interoperability among States |
| Indraratna *et al.* (2021)  (35) | Australia | Qualitative / RCT | Non-academic | Home | Patients with acute coronary syndrome or heart failure (N=164) | 6-month | App | Researcher made/ Standalone | Diagnosis | Effectiveness | To assist patients either diagnosed with Covid-19, or outpatients with HF who represent a high-risk population both in terms of hospital readmission, and severe morbidity or mortality from Covid-19 infection, The presence of hospital staff in case of receiving a red alert from the mobile application | Preventing the re-admission of hospitalized patients & preventing the aggravation of the disease during the Covid-19 epidemic & Reducing unnecessary visits to the hospital | Unavailability Bluetooth pulse oximeters |
| Hameed *et al.* (2021)  (36) | India | Quantitative /prospective single-center | Non-academic | Home | Patients with URS or PCNL (N=33) | 7-week | APP | Researcher made/ Standalone | Follow-up | Effectiveness and tracking | It helps in patient education, stent tracking, symptom tracking, automated notifications and change of appointments and early intervention in case of any clinical problems | SRS Management & Avoiding stent complications & Convenient stent removal & To reduce risk of ‘forgotten stents’ | Need to hardware (e.g. smartphone or tablet device) & Limitation language |
| Salim *et al.* (2021) (37) | Malaysia | Quantitative /Cohort | Non-academic | Clinic | Patients with asthma  (N=5) | 2-day | App | Researcher made/ Standalone | Follow-up | Self-management | Offering information and providing an accessible asthma action plan & Motivating and supporting improved medication adherence & Promoting behavior change through a reward system & Ease of use | Offering information & Providing an accessible asthma action plan & Motivating and supporting improved medication adherence & Promoting behavior change through a reward system & Ease of use | Need internet |
| Woong *et al.* (2021) (38) | Singapore | Quantitative /Cross sectional | Non-academic | Hospital | Patients with Covid-19 (N=10) | 16-day | App | Researcher made/ Standalone | Treatment | Effectiveness | To complete the informational and psychosocial needs of patients | To bridge the physical divide between the patient and the healthcare team & To attempt to address the patients’ needs holistically & To help overcome some of the barriers of contact isolation & Satisfying information needs | Requiring an initial electronic registration |
| Burkhardt *et al.* (2021)  (39) | USA | Quantitative /Cross sectional | Academic | University | Individuals  (N=63 ) | N/M | App | Researcher made/ Standalone | Follow-up | Development, self-monitoring, and data sharing | Supporting Covid-19 symptom self-monitoring and data sharing with appropriate public health agencies | Using FHIR & Interoperability with other health informatics systems such as electronic health record (EHR) | Authentication & Authorization |
| Blom *et al.* (2021) (40) | Germany | Quantitative /Survey Study | Academic | Population | Individuals  (N=3276) | 13-week | App | Researcher made/ Standalone | Follow-up | Potential barriers to adoption of tracing app | Effectiveness of app-based contact tracing to contain the Covid-19 pandemic | N/M | Lack of motivation & Low income |
| Fiol-DeRoque *et al.* (2021) (41) | Spain | Quantitative/ RCT | Academic | Hospital | HCWs >18 (N=482)  (intervention group (N=248)  & control group (n=234)) | 10-week | App | Researcher made/  N/M | Treatment | Effectiveness | Reduced mental health problems | Involving HCWs & Reducing posttraumatic stress, insomnia, anxiety, and stress | Need to hardware (e.g. smartphone) |
| Ang *et al.*  (2021) (42) | Singapore | Quantitative/ Before-after study | Non-academic | Clinic | Patients with type 2 diabetes (N=21) | 14-week | App | Researcher made/ Standalone | Treatment | To evaluate the feasibility, acceptability, and effectiveness | Cost- effectiveness & Significant reductions in HbA1c and body weight | Involving patients & Overcoming the challenges of chronic disease management including disruptions in face-to-face access to healthcare & Training patients for self-administration | Need to software & Need to hardware (e.g. smartphone and Abbott Freestyle Libre CGM device) & Need to in-person visit |
| Özkan *et al.*(2021) (43) | Turkey | Quantitative/Cross-sectional | Academic | Home | Pregnant women  (N=376) | 4-month | App | Premade/  N/M | Follow-up | To identify the use of mobile apps | Reduction in the receipt of face-to-face health services by pregnant women and their anxiety & Predicting the state of worry about the transmission of the Covid-19 and the level of pregnancy distress | Involving patients & Identify factors associated with pregnancy distress level | Need internet &  Need to up-to-date and evidence-based information |
| Campbell *et al.*  (2021) (44) | USA | Mixed-methods/ Cross-sectional | Academic | Clinic | Patients with HIV  (N=6668) | Pre-Covid-19 (N=2-month) & early Covid-19 (N= 1 month) | Message | N/A | Prevention | To evaluated secure messaging  and to investigate the content and function of the messages | Connect patients and providers through secure in-app messaging & identify needs & organize healthcare resources & strengthen patient care team relationships & Exchange messages | Sharing important information & Involving clinicians & Involving patients & Adaptable to low literacy & Accessible to people with lower economic and educational levels and/or members of minority racial/ethnic groups | Privacy & Data security |
| Zamberg *et al.*  (2020) (45) | Switzerland | Quantitative/Evaluation study | Academic | Hospital | HCWs  (N=125) | 2- week | App | Researcher made/ Standalone | Prevention | To disseminate up-to-date and validated information | Feeling confident in the medical staff & being informed about the methods of caring for patients with Covid-19 & less need to search for information sources | Effectiveness & Time-saving method for communicating | Need to hardware (e.g. iOS or Android phones) |
| Echeverría *et al.* (2020) (46) | Spain | Quantitative/Implementation Study | Academic | Hospital | Individuals  (N=10,347) &  HCWs  (N= 4000) | One month | App | Researcher made/ Integrated | Prevention & Follow-Up | Implementation | Early identification and self-isolation of suspected cases & Remote monitoring of mild cases & Real-time monitoring of the progression of the infection | Involving patients & Involving patient’s family & Limiting the risk of spreading the virus | Need to hardware (e.g. iOS or Android phones) & Security |
| Windisch *et al.* (2020) (47) | Switzerland | Quantitative/ Cross-sectional | Academic | Hospital | HCWs  (N= 1233) | 4-month | App | Researcher made/ Standalone | Prevention | An institutional knowledge dissemination strategy of COVID-19–related guidance to all HCWs | Increasing the reach of validated knowledge by HCWs & Significant increase in user activity | Involving HCWs | Need to hardware (e.g. iOS or Android phones) &  Need to up-to-date information |
| Gensheimer *et al.* (2020) (48) | Afghanistan | Quantitative /prospective case | Non-academic | Hospital | Medical professionals (N=48) | 4-month | App | Researcher made/ Standalone | Treatment | Assessment | Agreement between the teleophthalmology diagnosis and final diagnosis | Accuracy in disease diagnosis & Low response time & Preventing aeromedical evacuation & Secure and compliant consults | The complexity of diagnosing eye diseases with remote consultation |
| Timmers *et al.* (2020)  (49) | Netherlands | Quantitative /observational cohort | Non-academic | Home | Individuals  (N=6194) | 7-day | App | Researcher made/ Standalone | Prevention | Self-assessment and monitoring | To support people with Covid-19 education, self-assessment, and monitoring of their own health for a 7-day period by using a symptom diary | Self-assessment | Need to hardware (e.g. smart device) & Need to data sharing |
| Yasaka *et al.* (2020) (50) | USA | Mixed-methods/ Observational cohort study | Non-academic | Home | Individuals  (N=6194) | 14-day | App | Researcher made/ Standalone | Follow-up | Development | Self-report a positive Covid-19 status to their peer network | Increasing privacy & Demonstrating the potential to suppress an epidemic or pandemic outbreak & Improving adoption rates | Lack of motivation |
| Ben-Zeev *et al.* (2020)  (51) | USA | Quantitative / RCT | Non-academic | Home | Patients with SMIs  (N=315) | 30-day | App | Researcher made/ Standalone | Treatment | A smartphone intervention that comprises daily exercises designed to promote reassessment of dysfunctional beliefs in multiple domains. | Improving recovery and reducing the severity of psychiatric symptoms among individuals with SMIs | Easy to use & Sufficiently interactive & Effective in improving recovery & Reducing the severity of psychiatric symptoms | Need to hardware (e.g. smart device) & Need to data sharing |
| Thomas Foster Scherr *et al.* (2020) (52) | USA | Quantitative / Cross-sectional | Academic | University | Individuals  (N=45) | 6-week | App | Premade/  Standalone | Follow-up | Assessment | To identify persons at risk of Covid-19 | Increasing privacy | Inability to determine how long users stayed at a particular location |
| LaraAlbert *et al.* (2020) (53) | Spain | Quantitative / RCT | Non-academic | Home | Pregnant women with Gestational diabetes mellitus (GDM) (N=20) | 17-day | App | Researcher made/ Standalone | Treatment | Effectiveness | To prevent unnecessary hospital visits & To reduce clinicians’ workload for GDM management | Having decision support tools & More user-friendly & Easy to use | N/M |
| Badrick *et al.* (2020) (54) | Australia | Quantitative / Cross- sectional | Academic | Hospital | Patients with Covid-19 (N=15) | One week | App | Researcher made/ Standalone | Diagnosis | Development and deployment | To be as simple and error free as possible in the areas of registration, data entry, assessment, feedback, and supervisor monitoring | N/M | Need to hardware |
| Britt Elise Bente *et al.* (2020) (55) | Netherlands | Mixed-methods / Cross-sectional | Non-academic | Home | Individuals (N=44) | 4-day | App | Premade/Standalone | Follow-up | Tracking | Misconceptions about the app such as its usefulness and privacy-preserving mechanisms | Easy to use | Low level of education & Old age |

Covid-19: Coronavirus disease; RE: Role limitations due to personal or emotional problems, RF: Role limitations due to physical health problems, EF: Energy/fatigue, EW: Emotional well-being, SF: Social functioning, QoL: Quality of life; HCWs: Health Care Workers; SpO_2_: blood oxygen saturation; URS: Ureteric stent after ureteroscopy; PCNL: Percutaneous Nephrolithotomy; SMIs: Serious Mental Illnesses, SRS: Stent-Related Symptoms; FHIR: Fast Healthcare Interoperability Resources

^$^ The year of publication of the articles in the table is from old to new

N/A: not applicable N/M: not mention

1. Sun S, Lin D, Goldberg S, Shen Z, Chen P, Qiao S, et al. A mindfulness-based mobile health (mHealth) intervention among psychologically distressed university students in quarantine during the COVID-19 pandemic: A randomized controlled trial. Journal of counseling psychology. 2022;69(2):157.

2. Htet KKK, Phyu AN, Thwin T, Chongsuvivatwong V. Mobile Health App for Tuberculosis Screening and Compliance to Undergo Chest X-ray Examination Among Presumptive Cases Detected by the App in Myanmar: Usability Study. JMIR Formative Research. 2022;6(6):e37779.

3. Yoo PY, Movahed M, Rue I, Dos Santos CD, Majnemer A, Shikako K. Changes in Use of a Leisure Activity Mobile App for Children With Disabilities During the COVID-19 Pandemic: Retrospective Study. JMIR Pediatrics and Parenting. 2022;5(1):e32274.

4. Ha Y, Lee S-H, Lee D-H, Kang Y-H, Choi W, An J. Effectiveness of a Mobile Wellness Program for Nurses with Rotating Shifts during COVID-19 Pandemic: A Pilot Cluster-Randomized Trial. International Journal of Environmental Research and Public Health. 2022;19(2):1014.

5. Hodges J, Waselewski M, Harrington W, Franklin T, Schorling K, Huynh J, et al. Six-month outcomes of the HOPE smartphone application designed to support treatment with medications for opioid use disorder and piloted during an early statewide COVID-19 lockdown. Addiction science & clinical practice. 2022;17(1):1-11.

6. Mazaheri Asadi D. Mindfulness Training Intervention With the Persian Version of the Mindfulness Training Mobile App for Premenstrual Syndrome: A Randomized Controlled Trial. Frontiers in Psychiatry. 2022:1248.

7. Sadural E, Riley KE, Zha P, Pacquiao D, Faust A. Experiences With a Postpartum mHealth Intervention During the COVID-19 Pandemic: Key Informant Interviews Among Patients, Health Care Providers, and Stakeholders. JMIR formative research. 2022;6(6):e37777.

8. Pulik Ł, Romaniuk K, Dyrek N, Grabowska N, Łęgosz P. First Polish mobile application for patients undergoing total hip arthroplasty. Reumatologia/Rheumatology. 2022;60(3):224-8.

9. Quifer-Rada P, Aguilar-Camprubí L, Padró-Arocas A, Gómez-Sebastià I, Mena-Tudela D. Impact of COVID-19 Pandemic in Breastfeeding Consultations on LactApp, an m-Health Solution for Breastfeeding Support. Telemedicine and e-Health. 2022.

10. Akin-Sari B, Inozu M, Haciomeroglu AB, Trak E, Tufan D, Doron G. The daily use of a training using a mobile app as a coping tool against COVID-19 distress: A crossover randomized controlled trial. Journal of Affective Disorders. 2022.

11. Casalino G, Castellano G, Zaza G. Evaluating the robustness of a contact-less mHealth solution for personal and remote monitoring of blood oxygen saturation. Journal of Ambient Intelligence and Humanized Computing. 2022:1-10.

12. Marco-Ahulló A, Montesinos-Magraner L, González L-M, Crespo-Rivero T, Launois-Obregón P, García-Massó X, editors. Encouraging People with Spinal Cord Injury to Take Part in Physical Activity in the COVID-19 Epidemic through the mHealth ParaSportAPP. Healthcare; 2022: MDPI.

13. Alsaqer K, Bebis H. Self-care of hypertension of older adults during COVID-19 lockdown period: a randomized controlled trial. Clinical Hypertension. 2022;28(1):1-13.

14. Gonzalez-Ramirez M, Sanchez-Carrera R, Cejudo-Lopez A, Lozano-Navarrete M, Salamero Sánchez-Gabriel E, Torres-Bengoa MA, et al. Short-Term Pilot Study to Evaluate the Impact of Salbi Educa Nutrition App in Macronutrients Intake and Adherence to the Mediterranean Diet: Randomized Controlled Trial. Nutrients. 2022;14(10):2061.

15. Tseng CH, Chen R-J, Tsai S-Y, Wu T-R, Tsaur W-J, Chiu H-W, et al. Exploring the COVID-19 Pandemic as a Catalyst for Behavior Change Among Patient Health Record App Users in Taiwan: Development and Usability Study. Journal of Medical Internet Research. 2022;24(1):e33399.

16. Radotra I, Azimi DY, Maamoun W. The use of smartphone-application based medical photography for open fractures: A national survey of orthoplastic affiliated Major Trauma Centres in England. Injury. 2022;53(6):2028-34.

17. Alanzi TM, Althumairi A, Aljaffary A, Alfayez A, Alsalman D, Alanezi F, et al. Evaluation of the Mawid mobile healthcare application in delivering services during the COVID-19 pandemic in Saudi Arabia. International Health. 2022;14(2):142-51.

18. Gonzalez-Plaza E, Bellart J, Arranz Á, Luján-Barroso L, Crespo Mirasol E, Seguranyes G. Effectiveness of a Step Counter Smartband and Midwife Counseling Intervention on Gestational Weight Gain and Physical Activity in Pregnant Women With Obesity (Pas and Pes Study): Randomized Controlled Trial. JMIR Mhealth Uhealth. 2022;10(2):e28886.

19. Jobbágy A, Kiss N, Meznerics FA, Farkas K, Plázár D, Bozsányi S, et al. Emergency Use and Efficacy of an Asynchronous Teledermatology System as a Novel Tool for Early Diagnosis of Skin Cancer during the First Wave of COVID-19 Pandemic. International Journal of Environmental Research and Public Health [Internet]. 2022; 19(5).

20. Gasteiger N, Gasteiger C, Vedhara K, Broadbent E. The more the merrier! Barriers and facilitators to the general public’s use of a COVID-19 contact tracing app in New Zealand. Informatics for Health and Social Care. 2022;47(2):132-43.

21. Fukuti P, Uchôa CLM, Mazzoco MF, Cruz IDAGd, Echegaray MVF, Humes EdC, et al. COMVC-19: A Program to protect healthcare workers' mental health during the COVID-19 Pandemic. What we have learned. Clinics. 2021;76:e2631.

22. Hansen ST, Ehrari H, Kristiansen S, Olsen LS, Jensen RS, Kjær TW, et al. User perspectives and preferences regarding a mobile health cough application: A qualitative study during the coronavirus disease pandemic in Denmark. Digital Health. 2021;7:20552076211045590.

23. Mbiine R, Nakanwagi C, Lekuya HM, Aine J, Hakim K, Nabunya L, et al. An Early Warning Mobile Health Screening and Risk Scoring App for Preventing In-Hospital Transmission of COVID-19 by Health Care Workers: Development and Feasibility Study. JMIR Formative Research. 2021;5(12):e27521.

24. Golden EA, Zweig M, Danieletto M, Landell K, Nadkarni G, Bottinger E, et al. A resilience-building app to support the mental health of health care workers in the COVID-19 era: Design process, distribution, and evaluation. JMIR Formative Research. 2021;5(5):e26590.

25. Echeverría P, Puig J, Ruiz JM, Herms J, Sarquella M, Clotet B, et al. Remote Health Monitoring in the Workplace for Early Detection of COVID-19 Cases during the COVID-19 Pandemic Using a Mobile Health Application: COVIDApp. International Journal of Environmental Research and Public Health. 2021;19(1):167.

26. Magnani JW, Ferry D, Swabe G, Martin D, Chen X, Brooks MM, et al. Rurality and atrial fibrillation: a pathway to virtual engagement and clinical trial recruitment in response to COVID-19. American heart journal plus: cardiology research and practice. 2021;3:100017.

27. Hochstatter KR, Akhtar WZ, Dietz S, Pe-Romashko K, Gustafson DH, Shah DV, et al. Potential influences of the COVID-19 pandemic on drug use and HIV care among people living with HIV and substance use disorders: experience from a pilot mHealth intervention. AIDS and Behavior. 2021;25(2):354-9.

28. Hanson P, Summers C, Panesar A, Oduro-Donkor D, Lange M, Menon V, et al. Low Carb Program Health App Within a Hospital-Based Obesity Setting: Observational Service Evaluation. JMIR Formative Research. 2021;5(9):e29110.

29. Moulaei K, Sheikhtaheri A, Ghafaripour Z, Bahaadinbeigy K. The development and usability assessment of an mHealth application to encourage self-care in pregnant women against COVID-19. Journal of Healthcare Engineering. 2021;2021.

30. Anyanwu EC, Ward RP, Shah A, Arora V, Umscheid CA. A mobile app to facilitate socially distanced hospital communication during COVID-19: Implementation experience. JMIR mHealth and uHealth. 2021;9(2):e24452.

31. Siregar KN, Kurniawan R, BaharuddinNur RJ, Nuridzin DZ, Handayani Y, Halim L. Potentials of community-based early detection of cardiovascular disease risk during the COVID-19 pandemic. BMC Public Health. 2021;21(1):1-8.

32. Marshall JM, Dunstan DA, Bartik W. Smartphone Psychological Therapy During COVID-19: A Study on the Effectiveness of Five Popular Mental Health Apps for Anxiety and Depression. Frontiers in psychology. 2021;12:775775.

33. Vilendrer S, Amano A, Johnson CGB, Favet M, Safaeinili N, Villasenor J, et al. An app-based intervention to support first responders and essential workers during the COVID-19 pandemic: Needs assessment and mixed methods implementation study. Journal of medical Internet research. 2021;23(5):e26573.

34. Montanari Vergallo G, Zaami S, Marinelli E. The COVID-19 pandemic and contact tracing technologies, between upholding the right to health and personal data protection. Eur Rev Med Pharmacol Sci. 2021;25(5):2449-56.

35. Indraratna P, Biswas U, Yu J, Schreier G, Ooi S-Y, Lovell NH, et al. Trials and Tribulations: mHealth Clinical Trials in the COVID-19 Pandemic. Yearbook of Medical Informatics. 2021;30(01):272-9.

36. Hameed BZ, Shah M, Naik N, Reddy SJ, Somani BK. Use of ureteric stent related mobile phone application (UROSTENTZ App) in COVID-19 for improving patient communication and safety: a prospective pilot study from a university hospital. Cent European J Urol. 2021;74(1):51-6.

37. Salim H, Lee PY, Sharif-Ghazali S, Cheong AT, Wong J, Young I, et al. Developing an Asthma Self-management Intervention Through a Web-Based Design Workshop for People With Limited Health Literacy: User-Centered Design Approach. J Med Internet Res. 2021;23(9):e26434.

38. Woong NL, Ekstrom VSM, Xin X, Lim C, Boon ESK, Teo SWJ, et al. Empower to connect and connect to empower: experience in using a humanistic approach to improve patients’ access to, and experience of, care in isolation wards during the COVID-19 outbreak in Singapore. BMJ Open Quality. 2021;10(1):e000996.

39. Burkhardt HA, Brandt PS, Lee JR, Karras SW, Bugni PF, Cvitkovic I, et al. StayHome: A FHIR-Native Mobile COVID-19 Symptom Tracker and Public Health Reporting Tool. Online Journal of Public Health Informatics. 2021;13(1).

40. Blom AG, Wenz A, Cornesse C, Rettig T, Fikel M, Friedel S, et al. Barriers to the Large-Scale Adoption of a COVID-19 Contact Tracing App in Germany: Survey Study. J Med Internet Res. 2021;23(3):e23362.

41. Fiol-DeRoque MA, Serrano-Ripoll MJ, Jiménez R, Zamanillo-Campos R, Yáñez-Juan AM, Bennasar-Veny M, et al. A Mobile phone–based intervention to reduce mental health problems in health care workers during the COVID-19 pandemic (PsyCovidApp): randomized controlled trial. JMIR mHealth and uHealth. 2021;9(5):e27039.

42. Ang IYH, Tan KXQ, Tan C, Tan CH, Kwek JWM, Tay J, et al. A Personalized Mobile Health Program for Type 2 Diabetes During the COVID-19 Pandemic: Single-Group Pre–Post Study. JMIR Diabetes. 2021;6(3):e25820.

43. Özkan Şat S, Yaman Sözbir Ş. Use of mobile applications by pregnant women and levels of pregnancy distress during the COVID-19 (Coronavirus) pandemic. Maternal and Child Health Journal. 2021;25(7):1057-68.

44. Campbell BR, Swoger S, Tabackman A, Hilgart E, Elliott B, Coffey S, et al. PositiveLinks and the COVID-19 Response: Importance of Low-Barrier Messaging for PLWH in Non-urban Virginia in a Crisis. AIDS and Behavior. 2021;25(11):3519-27.

45. Zamberg I, Manzano S, Posfay-Barbe K, Windisch O, Agoritsas T, Schiffer E. A mobile health platform to disseminate validated institutional measurements during the COVID-19 outbreak: utilization-focused evaluation study. JMIR public health and surveillance. 2020;6(2):e18668.

46. Echeverría P, Bergas MAM, Puig J, Isnard M, Massot M, Vedia C, et al. COVIDApp as an innovative strategy for the management and follow-up of COVID-19 cases in long-term care facilities in Catalonia: implementation study. JMIR public health and surveillance. 2020;6(3):e21163.

47. Windisch O, Zamberg I, Zanella M-C, Gayet-Ageron A, Blondon K, Schiffer E, et al. Using mHealth to increase the reach of local guidance to health professionals as part of an institutional response plan to the COVID-19 outbreak: usage analysis study. JMIR mHealth and uHealth. 2020;8(8):e20025.

48. Gensheimer WG, Miller KE, Stowe J, Little J, Legault GL. Military Teleophthalmology in Afghanistan Using Mobile Phone Application. JAMA Ophthalmology. 2020;138(10):1053-60.

49. Timmers T, Janssen L, Stohr J, Murk JL, Berrevoets MAH. Using eHealth to Support COVID-19 Education, Self-Assessment, and Symptom Monitoring in the Netherlands: Observational Study. JMIR Mhealth Uhealth. 2020;8(6):e19822.

50. Yasaka TM, Lehrich BM, Sahyouni R. Peer-to-Peer Contact Tracing: Development of a Privacy-Preserving Smartphone App. JMIR Mhealth Uhealth. 2020;8(4):e18936.

51. Ben-Zeev D, Chander A, Tauscher J, Buck B, Nepal S, Campbell A, et al. A Smartphone Intervention for People With Serious Mental Illness: Fully Remote Randomized Controlled Trial of CORE. J Med Internet Res. 2021;23(11):e29201.

52. Scherr TF, Hardcastle AN, Moore CP, DeSousa JM, Wright DW. Understanding On-Campus Interactions With a Semiautomated, Barcode-Based Platform to Augment COVID-19 Contact Tracing: App Development and Usage. JMIR Mhealth Uhealth. 2021;9(3):e24275.

53. Albert L, Capel I, García-Sáez G, Martín-Redondo P, Hernando ME, Rigla M. Managing gestational diabetes mellitus using a smartphone application with artificial intelligence (SineDie) during the COVID-19 pandemic: Much more than just telemedicine. Diabetes research and clinical practice. 2020;169:108396.

54. Badrick T, Wienholt L, Fone D, Holzhauser D. The challenge of producing an EQA for the COVID-19 pandemic. Practical Laboratory Medicine. 2020;22:e00179.

55. Bente BE, van 't Klooster JWJR, Schreijer MA, Berkemeier L, van Gend JE, Slijkhuis PJH, et al. The Dutch COVID-19 Contact Tracing App (the CoronaMelder): Usability Study. JMIR Form Res. 2021;5(3):e27882.

/
