# Supplementary material for: Facilitators and barriers of mHealth interventions during the Covid-19 pandemic: systematic review
Source: BMC Health Serv Res. 2023 Oct 28;23:1176. doi: 10.1186/s12913-023-10171-w (PMC10613392; doi:10.1186/s12913-023-10171-w)
Supplement: Supplementary file 2 — Article Quality Assessment based on MMAT [file 12913_2023_10171_MOESM2_ESM.docx]

| Supplementary file 2. Article Quality Assessment based on MMAT | | | | | | | | | | | | | | | | | | | | | | | | | | | | | | | | | | | | | | | | | | | | | | | | | | | | | | | | |
| --- | --- | --- | --- | --- | --- | --- | --- | --- | --- | --- | --- | --- | --- | --- | --- | --- | --- | --- | --- | --- | --- | --- | --- | --- | --- | --- | --- | --- | --- | --- | --- | --- | --- | --- | --- | --- | --- | --- | --- | --- | --- | --- | --- | --- | --- | --- | --- | --- | --- | --- | --- | --- | --- | --- | --- | --- |
| **Domain** | **Criterion** | | **1** | **2** | **3** | | **4** | | **5** | | **6** | | **7** | **8** | | **9** | | **10** | | **11** | | **12** | | **13** | | **14** | | **15** | | **16** | | **17** | | **18** | | **19** | | **20** | | **21** | | **22** | | **23** | | **24** | | **25** | | **26** | | **27** | | **28** | **29** | **30** |
| **Screening questions** | Are there clear research questions? | | √ | √ | √ | | √ | | √ | | √ | | √ | √ | | √ | | √ | | √ | | √ | | √ | | √ | | √ | | √ | | √ | | √ | | √ | | √ | | √ | | √ | | √ | | √ | | √ | | √ | | √ | | √ | √ | √ |
|  | Do the collected data allow to address the research questions? | | √ | √ | √ | | √ | | √ | | √ | | √ | √ | | √ | | √ | | √ | | √ | | √ | | √ | | √ | | √ | | √ | | √ | | √ | | √ | | √ | | √ | | √ | | √ | | √ | | √ | | √ | | √ | √ | √ |
| **Qualitative** | Is the qualitative approach appropriate to answer the research question? | |  |  |  | |  | |  | |  | | √ | √ | |  | |  | |  | |  | |  | |  | |  | | √ | |  | |  | |  | |  | |  | | √ | |  | |  | |  | |  | |  | |  |  |  |
|  | Are the qualitative data collection methods adequate to address the research question? | |  |  |  | |  | |  | |  | | √ | √ | |  | |  | |  | |  | |  | |  | |  | | √ | |  | |  | |  | |  | |  | | √ | |  | |  | |  | |  | |  | |  |  |  |
|  | Are the findings adequately derived from the data? | |  |  |  | |  | |  | |  | | √ | √ | |  | |  | |  | |  | |  | |  | |  | | √ | |  | |  | |  | |  | |  | | √ | |  | |  | |  | |  | |  | |  |  |  |
|  | Is the interpretation of results sufficiently substantiated by data? | |  |  |  | |  | |  | |  | | √ | √ | |  | |  | |  | |  | |  | |  | |  | |  | |  | |  | |  | |  | |  | | √ | |  | |  | |  | |  | |  | |  |  |  |
|  | Is there coherence between qualitative data sources, collection, analysis and interpretation? | |  |  |  | |  | |  | |  | | √ | √ | |  | |  | |  | |  | |  | |  | |  | | √ | |  | |  | |  | |  | |  | | √ | |  | |  | |  | |  | |  | |  |  |  |
| **Quantitative randomized controlled (trials)** | Is randomization appropriately performed? | | √ |  |  | | √ | |  | | √ | |  |  | |  | | √ | |  | |  | | √ | | √ | |  | |  | |  | | √ | |  | |  | |  | |  | |  | |  | |  | | √ | |  | |  |  |  |
|  | Are the groups comparable at baseline? | | √ |  |  | | √ | |  | | √ | |  |  | |  | | √ | |  | |  | | √ | | √ | |  | |  | |  | | √ | |  | |  | |  | |  | |  | |  | |  | | √ | |  | |  |  |  |
|  | Are there complete outcome data? | | √ |  |  | | √ | |  | | √ | |  |  | |  | | √ | |  | |  | | √ | | √ | |  | |  | |  | | √ | |  | |  | |  | |  | |  | |  | |  | | √ | |  | |  |  |  |
|  | Are outcome assessors blinded to the intervention provided? | | √ |  |  | | √ | |  | | √ | |  |  | |  | | √ | |  | |  | | √ | | √ | |  | |  | |  | | √ | |  | |  | |  | |  | |  | |  | |  | | √ | |  | |  |  |  |
|  | Did the participants adhere to the assigned intervention? | | √ |  |  | | √ | |  | | √ | |  |  | |  | | √ | |  | |  | | √ | | √ | |  | |  | |  | | √ | |  | |  | |  | |  | |  | |  | |  | | √ | |  | |  |  |  |
| **Quantitative**  **non- randomized** | Are the participants representatives of the target population? | |  | √ | √ | |  | | √ | |  | |  |  | |  | |  | | √ | | √ | |  | |  | | √ | |  | | √ | |  | | √ | | √ | | √ | |  | | √ | | √ | | √ | |  | | √ | | √ | √ | √ |
|  | Are measurements appropriate regarding both the outcome and intervention (or exposure)? | |  | √ | √ | |  | | √ | |  | |  |  | |  | |  | | √ | | √ | |  | |  | | √ | |  | | √ | |  | | √ | |  | | √ | |  | | √ | | √ | | √ | |  | | √ | | √ | √ | √ |
|  | Are there complete outcome data? | |  | √ | √ | |  | | √ | |  | |  |  | |  | |  | | √ | | √ | |  | |  | | √ | |  | | √ | |  | | √ | | √ | | √ | |  | | √ | | √ | | √ | |  | | √ | | √ | √ | √ |
|  | Are the confounders accounted for in the design and analysis? | |  | √ | √ | |  | | √ | |  | |  |  | |  | |  | | √ | | √ | |  | |  | | √ | |  | | √ | |  | | √ | | √ | | √ | |  | | √ | | √ | |  | |  | | √ | | √ | √ | √ |
|  | During the study period, is the intervention administered (or exposure occurred) as intended? | |  | √ | √ | |  | |  | |  | |  |  | |  | |  | | √ | | √ | |  | |  | | √ | |  | | √ | |  | | √ | | √ | | √ | |  | | √ | | √ | | √ | |  | | √ | | √ |  | √ |
| **Quantitative descriptive** | Is the sampling strategy relevant to address the research question? | |  |  |  | |  | |  | |  | |  |  | | √ | |  | |  | |  | |  | |  | |  | |  | |  | |  | |  | |  | |  | |  | |  | |  | |  | |  | |  | |  |  |  |
|  | Is the sample representative of the target population? | |  |  |  | |  | |  | |  | |  |  | | √ | |  | |  | |  | |  | |  | |  | |  | |  | |  | |  | |  | |  | |  | |  | |  | |  | |  | |  | |  |  |  |
|  | Are the measurements appropriate? | |  |  |  | |  | |  | |  | |  |  | | √ | |  | |  | |  | |  | |  | |  | |  | |  | |  | |  | |  | |  | |  | |  | |  | |  | |  | |  | |  |  |  |
|  | Is the risk of nonresponse bias low? | |  |  |  | |  | |  | |  | |  |  | | √ | |  | |  | |  | |  | |  | |  | |  | |  | |  | |  | |  | |  | |  | |  | |  | |  | |  | |  | |  |  |  |
|  | Is the statistical analysis appropriate to answer the research question? | |  |  |  | |  | |  | |  | |  |  | | √ | |  | |  | |  | |  | |  | |  | |  | |  | |  | |  | |  | |  | |  | |  | |  | |  | |  | |  | |  |  |  |
| **Mixed methods** | Is there an adequate rationale for using a mixed methods design to address the research question? | |  |  |  | |  | |  | |  | |  |  | |  | |  | |  | |  | |  | |  | |  | |  | |  | |  | |  | |  | |  | |  | |  | |  | |  | |  | |  | |  |  |  |
|  | Are the different components of the study effectively integrated to answer the research question? | |  |  |  | |  | |  | |  | |  |  | |  | |  | |  | |  | |  | |  | |  | |  | |  | |  | |  | |  | |  | |  | |  | |  | |  | |  | |  | |  |  |  |
|  | Are the outputs of the integration of qualitative and quantitative components adequately interpreted? | |  |  |  | |  | |  | |  | |  |  | |  | |  | |  | |  | |  | |  | |  | |  | |  | |  | |  | |  | |  | |  | |  | |  | |  | |  | |  | |  |  |  |
|  | Are divergences and inconsistencies between quantitative and qualitative results adequately addressed? | |  |  |  | |  | |  | |  | |  |  | |  | |  | |  | |  | |  | |  | |  | |  | |  | |  | |  | |  | |  | |  | |  | |  | |  | |  | |  | |  |  |  |
|  | Do the different components of the study adhere to the quality criteria of each tradition of the methods involved? | |  |  |  | |  | |  | |  | |  |  | |  | |  | |  | |  | |  | |  | |  | |  | |  | |  | |  | |  | |  | |  | |  | |  | |  | |  | |  | |  |  |  |
| 1. Sun et al. 2022, 2. Htet et al. 2022, 3. Yoo et al. 2022, 4. Ha et al. 2022, 5. Hodges et al. 2022, 6. Mazaheri et al. 2022, 7. Sadural et al. 2022, 8. Pulik et al. 2022, 9. Quifer-Rada et al. 2022, 10. Akin-Sari et al. 2022, 11. Casalino et al. 2022, 12. Marco-Ahulló et al. 2022, 13. Alsaqer et al. 2022, 14. Gonzalez-Ramirez et al. 2022, 15. Tseng et al. 2022, 16. Radotra et al. 2022, 17. Alanzi et al. 2022, 18. Gonzalez-Plaza et al. 2022, 19. Jobbágy et al. 2022, 20. Gasteiger et al. 2022, 21. Fukuti et al. 2021, 22. Hansen et al. 2021, 23. Mbiine et al. 2021, 24. Golden et al. 2021, 25. Echeverría et al. 2021, 26. Magnani et al. 2021, 27. Hochstatter et al. 2021, 28. Hanson et al. 2021, 29. Moulaei et al. 2021, 30. Anyanwu et al. 2021 | | | | | | | | | | | | | | | | | | | | | | | | | | | | | | | | | | | | | | | | | | | | | | | | | | | | | | | | |
| Supplementary file 2. Article Quality Assessment based on MMAT (Continued) | | | | | | | | | | | | | | | | | | | | | | | | | | | | | | | | | | | | | | | | | | | | | | | | | | | | |  |  |  |  |
| **Domain** | | **Criterion** | **31** | **32** | | **33** | | **34** | | **35** | | **36** | | | **37** | | **38** | | **39** | | **40** | | **41** | | **42** | | **43** | | **44** | | **45** | | **46** | | **47** | | **48** | | **49** | | **50** | | **51** | | **52** | | **53** | | **54** | | **55** | |  |  |  |  |
| **Screening questions** | | Are there clear research questions? | √ | √ | | √ | | √ | | √ | | √ | | | √ | | √ | | √ | | √ | | √ | | √ | | √ | | √ | | √ | | √ | | √ | | √ | | √ | | √ | | √ | | √ | | √ | | √ | | √ | |  |  |  |  |
|  |  | Do the collected data allow to address the research questions? | √ | √ | | √ | | √ | | √ | | √ | | | √ | | √ | | √ | | √ | | √ | | √ | | √ | | √ | | √ | | √ | | √ | | √ | | √ | | √ | | √ | | √ | | √ | | √ | | √ | |  |  |  |  |
| **Qualitative** | | Is the qualitative approach appropriate to answer the research question? |  |  | |  | | √ | |  | |  | | |  | |  | |  | |  | |  | |  | |  | |  | |  | |  | |  | |  | |  | |  | |  | |  | |  | |  | |  | |  |  |  |  |
|  |  | Are the qualitative data collection methods adequate to address the research question? |  |  | |  | | √ | |  | |  | | |  | |  | |  | |  | |  | |  | |  | |  | |  | |  | |  | |  | |  | |  | |  | |  | |  | |  | |  | |  |  |  |  |
|  |  | Are the findings adequately derived from the data? |  |  | |  | | √ | |  | |  | | |  | |  | |  | |  | |  | |  | |  | |  | |  | |  | |  | |  | |  | |  | |  | |  | |  | |  | |  | |  |  |  |  |
|  |  | Is the interpretation of results sufficiently substantiated by data? |  |  | |  | | √ | |  | |  | | |  | |  | |  | |  | |  | |  | |  | |  | |  | |  | |  | |  | |  | |  | |  | |  | |  | |  | |  | |  |  |  |  |
|  |  | Is there coherence between qualitative data sources, collection, analysis and interpretation? |  |  | |  | | √ | |  | |  | | |  | |  | |  | |  | |  | |  | |  | |  | |  | |  | |  | |  | |  | |  | |  | |  | |  | |  | |  | |  |  |  |  |
| **Quantitative randomized controlled (trials)** | | Is randomization appropriately performed? |  |  | |  | |  | | √ | |  | | |  | |  | |  | |  | | √ | |  | |  | |  | |  | |  | |  | |  | |  | |  | | √ | |  | | √ | |  | |  | |  |  |  |  |
|  |  | Are the groups comparable at baseline? |  |  | |  | |  | | √ | |  | | |  | |  | |  | |  | | √ | |  | |  | |  | |  | |  | |  | |  | |  | |  | | √ | |  | | √ | |  | |  | |  |  |  |  |
|  |  | Are there complete outcome data? |  |  | |  | |  | | √ | |  | | |  | |  | |  | |  | | √ | |  | |  | |  | |  | |  | |  | |  | |  | |  | | √ | |  | | √ | |  | |  | |  |  |  |  |
|  |  | Are outcome assessors blinded to the intervention provided? |  |  | |  | |  | | √ | |  | | |  | |  | |  | |  | | √ | |  | |  | |  | |  | |  | |  | |  | |  | |  | | √ | |  | | √ | |  | |  | |  |  |  |  |
|  |  | Did the participants adhere to the assigned intervention? |  |  | |  | |  | | √ | |  | | |  | |  | |  | |  | | √ | |  | |  | |  | |  | |  | |  | |  | |  | |  | | √ | |  | | √ | |  | |  | |  |  |  |  |
| **Quantitative**  **non- randomized** | | Are the participants representatives of the target population? |  | √ | |  | |  | |  | | √ | | | √ | | √ | | √ | | √ | |  | | √ | | √ | |  | | √ | | √ | | √ | | √ | | √ | |  | |  | | √ | |  | | √ | |  | |  |  |  |  |
|  |  | Are measurements appropriate regarding both the outcome and intervention (or exposure)? |  | √ | |  | |  | |  | | √ | | | √ | | √ | | √ | | √ | |  | | √ | | √ | |  | | √ | | √ | | √ | | √ | |  | |  | |  | | √ | |  | | √ | |  | |  |  |  |  |
|  |  | Are there complete outcome data? |  | √ | |  | |  | |  | | √ | | | √ | | √ | | √ | |  | |  | | √ | | √ | |  | | √ | | √ | | √ | | √ | | √ | |  | |  | | √ | |  | | √ | |  | |  |  |  |  |
|  |  | Are the confounders accounted for in the design and analysis? |  | √ | |  | |  | |  | | √ | | | √ | | √ | | √ | | √ | |  | | √ | | √ | |  | | √ | | √ | | √ | | √ | | √ | |  | |  | | √ | |  | | √ | |  | |  |  |  |  |
|  |  | During the study period, is the intervention administered (or exposure occurred) as intended? |  | √ | |  | |  | |  | | √ | | | √ | | √ | | √ | | √ | |  | | √ | | √ | |  | |  | | √ | | √ | | √ | | √ | |  | |  | | √ | |  | | √ | |  | |  |  |  |  |
| **Quantitative descriptive** | | Is the sampling strategy relevant to address the research question? |  |  | |  | |  | |  | |  | | |  | |  | |  | |  | |  | |  | |  | |  | |  | |  | |  | |  | |  | |  | |  | |  | |  | |  | |  | |  |  |  |  |
|  |  | Is the sample representative of the target population? |  |  | |  | |  | |  | |  | | |  | |  | |  | |  | |  | |  | |  | |  | |  | |  | |  | |  | |  | |  | |  | |  | |  | |  | |  | |  |  |  |  |
|  |  | Are the measurements appropriate? |  |  | |  | |  | |  | |  | | |  | |  | |  | |  | |  | |  | |  | |  | |  | |  | |  | |  | |  | |  | |  | |  | |  | |  | |  | |  |  |  |  |
|  |  | Is the risk of nonresponse bias low? |  |  | |  | |  | |  | |  | | |  | |  | |  | |  | |  | |  | |  | |  | |  | |  | |  | |  | |  | |  | |  | |  | |  | |  | |  | |  |  |  |  |
|  |  | Is the statistical analysis appropriate to answer the research question? |  |  | |  | |  | |  | |  | | |  | |  | |  | |  | |  | |  | |  | |  | |  | |  | |  | |  | |  | |  | |  | |  | |  | |  | |  | |  |  |  |  |
| **Mixed methods** | | Is there an adequate rationale for using a mixed methods design to address the research question? | √ |  | | √ | |  | |  | |  | | |  | |  | |  | |  | |  | |  | |  | | √ | |  | |  | |  | |  | |  | | √ | |  | |  | |  | |  | | √ | |  |  |  |  |
|  |  | Are the different components of the study effectively integrated to answer the research question? | √ |  | | √ | |  | |  | |  | | |  | |  | |  | |  | |  | |  | |  | | √ | |  | |  | |  | |  | |  | | √ | |  | |  | |  | |  | | √ | |  |  |  |  |
|  |  | Are the outputs of the integration of qualitative and quantitative components adequately interpreted? | √ |  | | √ | |  | |  | |  | | |  | |  | |  | |  | |  | |  | |  | | √ | |  | |  | |  | |  | |  | | √ | |  | |  | |  | |  | | √ | |  |  |  |  |
|  |  | Are divergences and inconsistencies between quantitative and qualitative results adequately addressed? | √ |  | | √ | |  | |  | |  | | |  | |  | |  | |  | |  | |  | |  | | √ | |  | |  | |  | |  | |  | | √ | |  | |  | |  | |  | | √ | |  |  |  |  |
|  |  | Do the different components of the study adhere to the quality criteria of each tradition of the methods involved? | √ |  | | √ | |  | |  | |  | | |  | |  | |  | |  | |  | |  | |  | | √ | |  | |  | |  | |  | |  | |  | |  | |  | |  | |  | | √ | |  |  |  |  |
| 31. Siregar et al. 2021, 32. Marshall et al. 2021, 33. Vilendrer et al. 2021, 34. Montanari Vergallo et al. 2021, 35. Indraratna et al. 2021, 36. Hameed et al. 2021, 37. Salim et al. 2021, 38. Woong et al. 2021, 39. Burkhardt et al. 2021, 40. Blom et al. 2021, 41. Fiol-DeRoque et al. 2021, 42.  Ang et al. 2021, 43. Özkan et al. 2021, 44. Campbell et al. 2021, 45. Zamberg et al. 2020, 46. Echeverría et al. 2020, 47. Windisch et al. 2020, 48. Gensheimer et al. 2020, 49. Timmers et al. 2020, 50. Yasaka et al. 2020, 51. Ben-Zeev et al. 2020, 52. Thomas Foster Scherr et al. 2020, 53. LaraAlbert et al. 2020, 54. Badrick et al. 2020, 55. Britt Elise Bente et al. 2020 | | | | | | | | | | | | | | | | | | | | | | | | | | | | | | | | | | | | | | | | | | | | | | | | | | | | |  |  |  |  |
